# Deprescribing: A Prime Opportunity to Optimize Care of Cancer Patients

**DOI:** 10.3390/curroncol30110704

**Published:** 2023-11-02

**Authors:** Genevieve Chaput, Hitesh Bhanabhai

**Affiliations:** Division of Supportive and Palliative Care, McGill University Health Centre, Department of Family Medicine, McGill University, Montreal, QC H4A 3J1, Canada

**Keywords:** deprescribing, goals of care, polypharmacy, advance care planning, comorbidities, patient-centered care

## Abstract

Patients with incurable cancers have an increasing number of comorbidities, which can lead to polypharmacy and its associated adverse events (drug-to-drug interaction, prescription of a potentially inappropriate medication, adverse drug event). Deprescribing is a patient-centered process aimed at optimizing patient outcomes by discontinuing medication(s) deemed no longer necessary or potentially inappropriate. Improved patient quality of life, risk reduction of side effects or worse clinical outcomes, and a decrease in healthcare costs are well-documented benefits of deprescribing. Deprescribing and advance care planning both require consideration of patients’ values, preferences, and care goals. Here, we provide an overview of comorbidities and associated polypharmacy risks in cancer patients, as well as useful tools and resources for deprescribing in daily practice, and we shed light on how deprescribing can facilitate advance care planning discussions with patients who have advanced cancer or a limited life expectancy.

## 1. Introduction

Cancer patients are often afflicted with other chronic comorbid conditions [1]. As pharmacological agents are routinely required in the management of comorbid conditions, polypharmacy is often observed in patients afflicted with cancer [2,3]. While clinical guidelines to managing comorbid conditions can promote the use of more than one medication to optimize patient outcomes, polypharmacy puts patients at increased risk of experiencing a drug-to-drug interaction [4], being prescribed a potentially inappropriate medication, or developing an adverse drug event [5,6]. More specifically, in advanced cancer and palliative care settings, unpredictable variations in medication pharmacokinetics and pharmacodynamics can occur, leading to decreased medication tolerance and burdensome side effects, which can be mistakenly believed to be due to the underlying disease [7]. Deprescribing, defined as the systematic process of identifying and discontinuing medications in circumstances in which existing or potential harms are greater than existing or potential benefits [8], can serve as a pivotal teaching moment in clinical practice by which to initiate, explore, or reassess the goals of care with respect to advanced cancer patients [9]. As the process of deprescribing requires the consideration of a patient’s care goals, values, preferences, functional level, and expected prognosis [8], advance care planning should be an integral part of deprescribing discussions with these patients [9]. This article provides an overview of the comorbidities and associated polypharmacy risks in the cancer population, as well as the available tools and resources designed to aid deprescribing in clinical practice, and illustrates how deprescribing may facilitate the initiation of advance care planning goals with patients afflicted with advanced cancer or with a shortened life expectancy.

## 2. Discussion

### 2.1. Comorbidities and Associated Polypharmacy Risks in Cancer Patients

An advanced or incurable cancer diagnosis can create a shift in chronic disease management and in the overarching goals of care. Common comorbidities in older cancer patients, defined as aged 65 and older, include cardiac (i.e., heart failure, ischemic heart disease) and pulmonary conditions (i.e., chronic obstructive pulmonary disease), chronic kidney disease, hypertension, and diabetes [10]. In addition to their respective morbidity and mortality risks, comorbidities further increase the risk of treatment-related toxicity and cancer-related morbidity and mortality [11]. As cancer patients with underlying comorbid conditions are more vulnerable, the establishment of risk-stratified, individualized care plans for these patients are of the utmost importance.

The management of comorbidities in cancer patients must carefully consider expected benefits versus harms. Moreover, treating each comorbidity as per its respective clinical guidelines creates unrealistic care plans that are not patient-centered and lead to polypharmacy [12]. Polypharmacy is associated with considerable risks for patients [4,5,6,13,14,15,16,17]. First, polypharmacy increases the risk of being prescribed potentially inappropriate medications, i.e., those for which the possible adverse effects are greater than the expected benefits; these are associated with higher risk of unplanned hospitalizations and decreased quality of life [18,19,20]. Discontinuation of medications such as antihypertensives and dyslipidemia agents, once appropriately prescribed for primary or secondary prevention purposes, may thus be pertinent in advanced cancer patients with a limited life expectancy (i.e., an estimated prognosis of 6 months or less) [21,22].

Moreover, potential drug-to-drug interactions are common in cancer patients. Approximately 25% to 70% of those undergoing oral or intravenous oncological treatments or receiving solely supportive care are deemed to be at risk of such interactions [4,23,24,25,26]. In addition to complex pharmacological profiles, cancer patients are at increased risk of drug-to-drug interactions due to changes in distribution volume in the presence of nutritional deficiency or edema, impaired medication absorption (e.g., mucositis), or excretion (e.g., underlying kidney or liver dysfunction) [24]. Medications associated with potential drug-to-drug interactions with oncological treatment regimens include selective serotonin reuptake inhibitors (e.g., (es)citalopram, sertraline, fluoxetine), anticonvulsants (e.g., phenytoin), non-steroidal anti-inflammatory drugs (e.g., aspirin, ibuprofen, naproxen), and opioids such as fentanyl [24,25]. Table 1 outlines potential drug-to-drug interactions associated with cancer treatment regimens [1].

Lastly, in the presence of polypharmacy, oncology patients appear to be more vulnerable to cancer-related adverse drug events. As an example, previous findings have revealed that a greater number of concomitant medications in patients receiving irinotecan was associated with increased irinotecan-related toxicity (e.g., neutropenia, diarrhea) [27]; more specifically, compared to patients receiving zero to one concomitant medication, those receiving four or more of the following concomitant medications experienced greater irinotecan-related toxicities: famotidine, ferrous sulfate, rabeprazole, amlodipine, benzbromarone, ranitidine, furosemide, spironolactone, lansoprazole, and/or olmesartan [27]. Additionally, as the majority of cancer patients aged 65 or older have underlying comorbid conditions requiring medications, and as cancer management typically includes chemotherapy or other adjunct or supportive pharmacotherapies, older age may also increase the risk of adverse drug reactions in cancer patients [28]. These findings further highlight the importance of routine medication profile assessments and the deprescription of pharmacological agents when potential risks outweigh previously anticipated benefits.

### 2.2. Deprescribing in Oncology Practice: An Essential Component of Care

Deprescribing is defined as a patient-centered process which aims to improve patient outcomes by discontinuing medication(s) that may no longer be necessary or which may be potentially inappropriate [29]. Although an integral part of good prescribing practices, deprescribing has yet to become a gold-standard practice, with studies showing the use of potentially inappropriate medications persisting in 22 to 95% of patients with advanced cancer [30,31]. The benefits of deprescribing are well-established and include an increase in patient quality of life [32,33], a reduction in the risk of side effects or worse clinical outcomes [5,34], and a decrease in healthcare costs [35,36,37].

For patients with advanced disease and reduced life expectancy, providers’ clinical reasoning about each pharmacological agent should shift from “how much will it help?” to “when will it help?” to guide their assessment of potential benefits versus harms [38]. Deprescribing guidelines and tools can facilitate medication cessation in oncology practices [39]. Based on patient characteristics (e.g., geriatric, advanced cancer, or estimated prognosis less than 6 months), providers can select one tool over another to aid in the deprescribing process [39]. As an example, the OncPal, a validated tool targeting cancer patients with a life expectancy of less than 6 months, offers deprescribing guidance on eight classes of medications [40]. In addition, brief deprescribing methods, such as the “6-Step method” and the “Steps to deprescribe”, can be easily integrated into patient reassessments [41,42].

Ideally, these step-by-step methods should be carried out regularly and, more particularly, during care transitions such as the shift from curative to palliative care goals, the failed response to a first-line palliative treatment regimen, or the cessation of active oncological treatments in the context of disease progression or worsening functional baseline. Table 2 provides a summary of deprescribing tools and guidelines [39]. When identifying more than one potentially inappropriate medication(s), deprescribing in succession is recommended [40]. Lastly, an interdisciplinary approach, including the involvement of a pharmacist, may also contribute to the optimization of patient care [43].

While barriers have been identified in the literature, several facilitators at the patient, caregiver, provider, and organizational levels can be leveraged when exploring potential medication cessation with patients [44]. Examples of patient facilitators include medication burden, psychological benefit of medication cessation, and overall distaste in medication [44]. Identification of such facilitators can aid providers in initiating deprescribing discussions and further elicit their patients’ values and understanding of their overall health and care goals [44]. Table 3 outlines facilitators and barriers to deprescribing [44]. Undoubtedly, shared decision making between patient and professional is a cornerstone of all deprescribing discussions, allowing for the assessment of potential benefits and harms of each medication, optimal patient engagement, and the best possible mutual comprehension of overarching priorities in the context of advanced cancer care delivery.

### 2.3. Deprescribing Discussions: An Opportunity for Advance Care Planning

Advance care planning, which aims to ensure that patients receive care reflecting their values and preferences, is an essential component of high-quality care for advanced cancer patients [45,46,47]. The benefits of advance care planning discussions are well-known and include greater patient autonomy, decreased length and number of hospitalizations, and reduced unwanted and unnecessary treatments [48,49]. Although recognized as the gold standard for cancer patients with a shortened life expectancy, advance care planning is infrequently undertaken and typically introduced late by physicians, with studies revealing these activities documented in less than 10% of advanced cancer patients [50,51,52,53,54]. Provider-level barriers to these discussions include personal discomfort with advance care planning and death, fear of crushing patients’ hope, and perceived insufficient experience in communicating about the goals of care and end-of-life planning [49,55]. Discussing deprescribing with patients can help providers initiate broader conversations about advance care planning, including goals of care.

Deprescribing and advance care planning discussions share similarities in that they both involve understanding patient and caregiver values, preferences, care goals, and life expectancy [8,9]. The process of deprescribing provides an opportunity to explore a patient’s understanding of the disease trajectory and to explain the potential trade-offs between prioritizing life prolongation versus comfort-focused care. A commonly encountered clinical case to illustrate this is that of the elderly patient with metastatic lung cancer who has experienced significant involuntary weight loss with progressive anorexia–cachexia. This patient, who remains on antihypertensive medications previously beneficial to control her high blood pressure, is now experiencing orthostatic hypotension and presyncope episodes. Such clinical presentation should not only prompt physicians to initiate a discussion about stopping some or all antihypertensive medications; it should also serve as an opportunity to gently converse with the patient about their declining health and engage in discussions about the overarching goals of care more likely to improve symptom burden and quality of life in the context of progressive and terminal malignancy.

Moreover, expected prognosis and medication time to benefit, which are fundamental in assessing medication appropriateness [56], may also facilitate conversations about goals of care. For example, in the case of an elderly man known for dyslipidemia on a statin for primary prevention faced with progressive metastatic pancreatic cancer, the patient’s reaction to the recommendation of statin discontinuation can inform providers of his understanding of their overall health and care goal preferences. In the context of incurable advanced pancreatic cancer, the estimated prognosis is likely less than one year, and more likely a few months, with or without systemic therapy, suggesting that cessation of the statin is logical and clinically appropriate [33]. However, patients’ perceptions are not always aligned with the biology of their disease, even in cases of irreversible cancer trajectories [57]. Thus, deprescribing discussions offer an opportunity to explore patients’ understanding of their health status by reviewing medication indications, including expected time to benefit, which may, in turn, facilitate broader discussions about goals of care in the context of advanced cancer.

Lastly, deprescribing discussions are also of utmost importance for patients receiving palliative chemotherapy, particularly in the presence of decreasing tolerance to regimen, worsening symptom burden, and declining functional status. Continuation of aggressive treatments in patients with poor prognoses lead to decreased patient quality of life, suboptimal resource utilization, and increased healthcare costs [49]. For those patients, frequent reassessments of palliative oncological treatment indications, as well as open discussions with patients about associated potential benefits versus harms, are warranted, reflecting good prescribing practices [29]. Such discussions can further facilitate the transition to more clinically appropriate care goals interventions, including the provision of supportive and palliative care services.

## 3. Conclusions

Deprescribing is part of good prescribing practices in cancer care. Deprescribing reduces risk of drug-to-drug interactions and adverse reactions and improves patients’ overall quality of life. Moreover, discussions surrounding medication cessation offers providers a prime opportunity to further explore advance care planning and care goals with patients, invaluable components of high-quality care delivery. By routinely engaging in both deprescribing and advance care planning discussions with patients, oncology providers will come closer to reaching the optimal win–win scenario, wherein patients receive the highest standard of care and healthcare resources are utilized more judiciously and efficiently.

## Figures and Tables

**Table 1:** Potential drug-to-drug interactions involving anticancer treatment (reprinted with permissions) [1].

Drug-Drug Combination(s)	Description of Interaction
Tamoxifen + ondansetron/granisetron/sotalol/erythromycin/levofloxacin/azithromycin Coumarins + capecitabine/tamoxifen/etoposide/carboplatin/paclitaxel/gemcitabine Methotrexate + sulfamethoxazole/trimethoprim/aspirin Phenytoin þ irinotecan (Es)omeprazole + dasatinib/nilotinib NSAIDs + corticosteroids/SSRIs/dipyridamole/clopidogrel/alendronate SSRIs + metoclopramide/tramadol Fentanyl + fluconazole/aprepitant/ketoconazole/diltiazem/itraconazole	Drug combinations can prolong QT interval Increased coumarin effect, bleeding may occur Increased methotrexate effect, increased bone marrow and hepatic toxicity Reduced irinotecan efficacy Proton pump inhibitors may decease plasma concentration of tyrosine kinase inhibitors Increased risk of gastrointestinal bleeding Risk of serotonin syndrome Increased fentanyl effects

NSAIDs, non-steroidal anti-inflammatory drugs, e.g., aspirin, diclofenac, ibuprofen, meloxicam, naproxen; SSRIs, selective serotonin reuptake inhibitors, e.g., (es)citalopram, fluoxetine, paroxetine, venlafaxine.

**Table 2:** Summary of deprescribing tools and guidelines identified (reprinted with permissions) [39].

Tool	Description	Target Population during Development
OncPal [10]	Validated against an expert opinion panel in a single-center study. It includes medications with a limited benefit in palliative cancer patients. It consists of eight medication classes: anticoagulants, cardiovascular agents, osteoporosis medications, peptic ulcer prophylaxis, oral hypoglycemics, vitamins, minerals, and complementary–alternative medicines.	Palliative cancer patients with a life expectancy <6 months
6-Step method [11]	A systematic method for deprescribing consisting of six steps: Step 0: Reappraisal of the patient’s clinical situation, setting treatment goals; Step 1: Finding out all the medications a patient is taking; Step 2: Agreement with patient and carers; Step 3: Identify drugs that can be deprescribed in the first place without causing harm; Step 4: Address medication that requires a long time until benefit, outside of the patients’ expected lifespan; Step 5: Identification of medications that could be withdrawn, but slowly; Step 6: Monitor carefully to identify clinical problems.	Advanced cancer patients
Steps to deprescribe [12]	A periodically carried out comprehensive medication assessment following five steps to deprescribe: Step 1: Reconcile all medications and consider indications; Step 2: Consider overall risk of harm; Step 3: Assess each drug in terms of current or future benefit in relation to current or future harm; Step 4: Prioritize drugs for deprescribing, giving preference to those that have the most unfavorable risk/benefit ratio and least likelihood of withdrawal symptoms; Step 5: Implement a discontinuation plan and monitor.	Older patients with cancer
Futility criteria by Oliveira et al. [13]	Criteria for futility for 7 medication categories, criteria modified from Fede et al. [14]. Medication categories included conditions for futility. Medication categories covered include gastric protectors, antihypertensive drugs, antidiabetic drugs, statins, anticoagulants, bisphosphonates, and antidementia drugs.	Advanced cancer patients with a life expectancy <6 months
Preventative medications by Todd et al. [15]	Classes of the most common inappropriate preventative medication in patients with life-limiting illnesses based on a systematic review: vitamins and minerals, antidiabetic, antihypertensive, antihyperlipidemic, and antiplatelet medications.	Patients with a life-limiting illnesses
Medications for chronic diseases by Garfinkel et al. [16]	Medications for chronic diseases. Topical preparations and drugs for oncological treatments were excluded (oral and/or intravenous cytostatic drugs and biological agents).	End-stage cancer patients referred to homecare hospice
Beers criteria [17]	PIMs to be avoided by older adults in most circumstances or under specific situations, updated by the American Geriatrics Society.	Geriatric population
STOPP criteria [18]	A screening tool of older people’s prescription (STOPP) criteria which consists of 80 criteria. These medications are associated with adverse drug events and can be used for older people.	Older patients
Medication appropriateness index [19]	A questionnaire of 10 questions used by physicians to fill in a score to assess if the use of a certain drug is appropriate or inappropriate. Questions are focused on, e.g., indications, dosage, durations, interactions, and effectiveness.	Ambulatory elderly patients

**Table 3:** Facilitators and barriers to deprescribing (reprinted with permissions) [44].

	Facilitators	Barriers
Patient and/or family/caregiver-level	General dislike of taking medications; Perceived lack of appropriateness of medication(s); Reduction in medication burden; Unsure about continued need; Experiencing medication side effects or adverse events; Psychological benefits of cessation; Cost; Fear of addiction/dependency; Considering alternative treatment options; Mistrust of prescriber who started medication; Knowledge that medications can be restarted if necessary; Follow-up visits by and support of prescribers; Other family or process support; Influenced to discontinue by other health care providers, family, or friends.	Perceived appropriateness of medications; Fear of medication withdrawal effects of return of condition; Lack of prescriber support/time; Need for appropriate timing for cessation; Previous bad experiences with stopping; Influenced to continue from other health care providers, family, or friends; Not wanting to have one’s mind occupied with tapering; Guilt related to depriving loved ones of something that might have worked; Insufficient reimbursement and/or resources.
Prescriber-level	Review, observation, audit, and feedback Devolve responsibility; Fear of negative consequences of continuation; Positive attitude toward deprescribing; Stopping brings benefits; Data to quantify benefit/harms; Dialogue with patients; Access to specialists; Confidence; Work experience, skills, and training; Monitoring by authorities; Stimulus to review; Adequate reimbursement; Access to support services; Patient receptivity/motivation to change; Patient poor prognosis.	Poor insight into deprescribing implementation; Discrepant beliefs and practice; Fear unknown/negative consequences of change; Drugs work, few side effects; Prescribing is kind, meets needs; Stopping is difficult, futile, has/will fail; Stopping is a lower-priority issue; Devolve responsibility; Skills/knowledge gaps; Lack of evidence; Incomplete clinical picture; Other health professionals; Patient ambivalence/resistance to change; Poor acceptance of alternatives; Difficult and intractable adverse circumstances; Discrepant goals to prescriber; Time and effort; Insufficient reimbursement; Limited availability of effective alternatives; Prescribe without review; Respect prescriber’s right to autonomy; Health care culture to prescribe more; Prescribing validates illness.
Organizational (System)-level	National health care system; Nursing home or palliative or hospice care setting; Pharmacist-led interventions; Computerized decision support systems; Support from medical, nursing, and/or pharmacy leadership.	Staff shortages; Lack of easily accessible deprescribing-focused evidence-based guidelines, tools, and resources.
